# How can we improve the access of forcibly displaced people to mental health care in Europe?

**DOI:** 10.1192/j.eurpsy.2025.129

**Published:** 2025-08-26

**Authors:** M. Schouler-Ocak

**Affiliations:** Psychiatry and Psychotherapy, Charité - Universitätsmedizin Berlin, Berlin, Germany

## Abstract

**Abstract:**

A growing body of literature report, that vulnerable groups eg refugee, asylum seeker and forcibly displaced groups across Europe face social and psychological challenges linked to their minority status, often involving discrimination and racism. Moreover, they have to overcome many other barriers to accessing healthcare and preventive interventions. There is increasing evidence that a large proportion of refugees or forcibly displaced persons suffer from the consequences of traumatic events and exhibit psychological problems or develop mental disorders, including post-traumatic stress disorder, depressive and anxiety disorders, and relapses in psychotic episodes. European countries are aware that psychosocial and health services face major challenges and need to develop or expand strategies to overcome them. The direct and indirect consequences of humanitarian catastrophes cannot be estimated at present. In this presentation, strategies to improve the access of forcibly displaced people to mental health care services will be presented and discussed.

**Disclosure of Interest:**

None Declared

